# Crystal structure of TcpK in complex with *oriT* DNA of the antibiotic resistance plasmid pCW3

**DOI:** 10.1038/s41467-018-06096-2

**Published:** 2018-09-13

**Authors:** Daouda A. K. Traore, Jessica A. Wisniewski, Sarena F. Flanigan, Paul J. Conroy, Santosh Panjikar, Yee-Foong Mok, Carmen Lao, Michael D. W. Griffin, Vicki Adams, Julian I. Rood, James C. Whisstock

**Affiliations:** 1 Department of Biochemistry and Molecular Biology, Infection and Immunity Program, Monash Biomedicine Discovery Institute, Monash University, Clayton, 3800 VIC Australia; 2 Faculté des Sciences et Techniques, Université des Sciences Techniques et Technologiques de Bamako (USTTB), BP E3206 Bamako, Mali; 3 Department of Microbiology, Infection and Immunity Program, Monash Biomedicine Discovery Institute, Monash University, Clayton, 3800 VIC Australia; 4 Australian Synchrotron, Clayton, 3168 VIC Australia; 5 Biochemistry and Molecular Biology, Bio21 Molecular Science and Biotechnology Institute, University of Melbourne, Parkville, 3010 VIC Australia; 6 ARC Centre of Excellence in Advanced Molecular Imaging, Monash University, Clayton, 3800 VIC Australia; 7 EMBL Australia, Monash University, Clayton, 3800 VIC Australia

## Abstract

Conjugation is fundamental for the acquisition of new genetic traits and the development of antibiotic resistance in pathogenic organisms. Here, we show that a hypothetical *Clostridium perfringens* protein, TcpK, which is encoded by the tetracycline resistance plasmid pCW3, is essential for efficient conjugative DNA transfer. Our studies reveal that TcpK is a member of the winged helix-turn-helix (wHTH) transcription factor superfamily and that it forms a dimer in solution. Furthermore, TcpK specifically binds to a nine-nucleotide sequence that is present as tandem repeats within the pCW3 origin of transfer (*oriT*). The X-ray crystal structure of the TcpK–TcpK box complex reveals a binding mode centered on and around the β-wing, which is different from what has been previously shown for other wHTH proteins. Structure-guided mutagenesis experiments validate the specific interaction between TcpK and the DNA molecule. Additional studies highlight that the TcpK dimer is important for specific DNA binding.

## Abstract (web summary)

Conjugative transfer of antibiotic resistance plasmid pCW3 in *Clostridium perfringens* is mediated by the *tcp* locus. Here, the authors identify a wHTH-type protein, TcpK, that is essential for efficient plasmid transfer and interacts with the plasmid *oriT* region in a unique manner.

## Introduction

In the Gram-positive pathogenic bacterium *Clostridium perfringens* the conjugative tetracycline resistance plasmid pCW3 is representative of a large class of closely related toxin and antibiotic resistance plasmids^1^. Conjugative transfer of this plasmid is mediated by the highly conserved *tcp* locus, which encodes at least eight proteins that are known to be essential for efficient DNA transfer^2^. Functionally, the conjugative machinery is known to be homologous, at least in part, to the type 4 secretion systems (T4SS) characterized in Gram-negative bacteria.

**Fig. 1:**
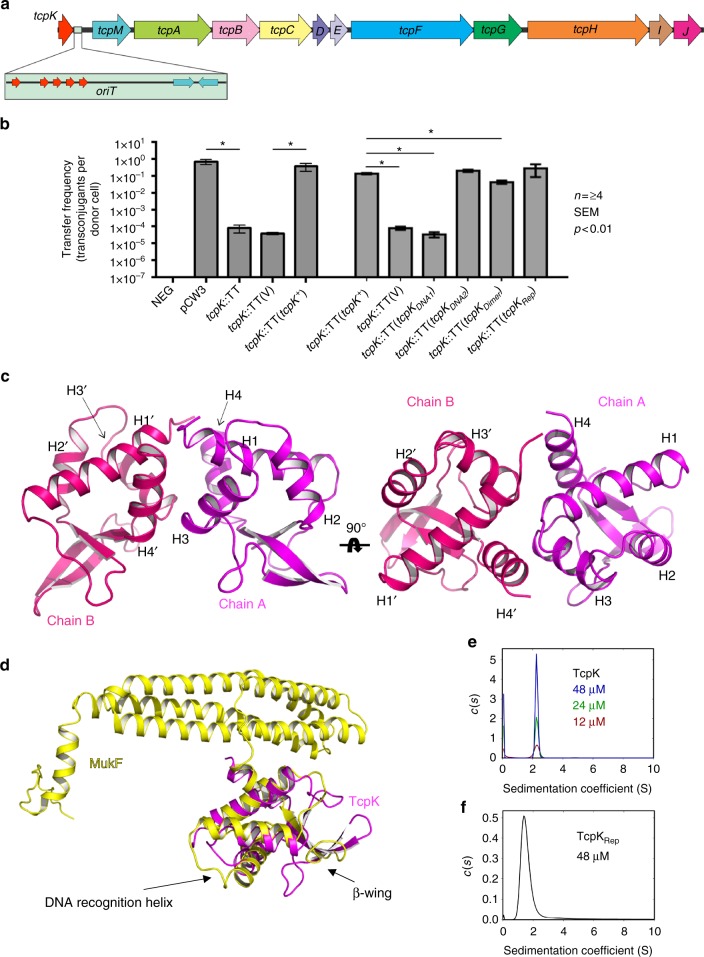
TcpK is a DNA binding protein required for efficient conjugative transfer of pCW3. **a** Genes encoded by the conjugation locus of pCW3. TcpK is located upstream of the *oriT* site. Direct repeats (red) and inverted repeats (blue) in the *oriT* site are shown in the insert. **b** Transfer frequencies of the pCW3 *tcpK* mutant and its complemented derivatives or site-directed mutants. The transfer frequencies are shown as the number of transconjugants per donor cell. Isogenic donor strains with a JIR325 background are shown on the *X*-axis. NEG non-conjugative tetracycline resistance plasmid pJIR1909 (JIR4493), pCW3 wild-type pCW3 (JIR4195), *tcpK*::TT pCW3*tcpK*::TT mutant (JIR13063), (V) JIR13063 with vector control plasmid (pJIR3422), TcpK^+^ JIR13063 with vector plasmid carrying wild-type *tcpK* (pJIR4411), *tcpK*_DNA1_, *tcpK*_DNA2_, *tcpK*_Dimer_ and *tcpK*_Rep_ JIR13063 with vectors encoding mutant *tcpK* derivatives (plasmids pJIR4581, pJIR4579, pJIR4577 and pJIR4580 respectively). The mean ± SEM values are based on results from at least four biological replicates. Statistical analysis was carried out using the Mann–Whitney test. Asterisk denotes statistical significance (*P* < 0.01) in comparison to pCW3 wild-type (positive control) or *tcpK*^*+*^ complementation strain. **c** Ribbon representation of the crystal structure of TcpK (dimer). Monomeric subunits are color coded differently and the helices are numbered H1 to H4 from N to C terminus. The pictures represent two orthogonal views along the horizontal axis. **d** Superposition of TcpK (magenta) with the full length MukF (yellow, PDB 3EUH). **e** Sedimentation velocity analysis of TcpK. Continuous sedimentation coefficient (*c*(*s*)) distribution for 12, 24, and 48 μM TcpK calculated from the best fit to sedimentation data shown in Supplementary Figure 3a–c. **f** Continuous sedimentation coefficient (*c*(*s*)) distribution for 48 μM TcpK_Rep_ calculated from the best fit to the sedimentation data shown in Supplementary Figure 3f

The *tcp* locus comprises 11 genes (*tcpA–J* and *tcpM*^2^, Fig. 1a). The core complex of the transfer apparatus is formed by the membrane proteins TcpH and TcpC (Supplementary Figure 1), which are homologous to the T4SS proteins VirB6 and VirB8, respectively^3–5^. TcpM functions as a novel relaxase and is responsible for initial processing of the plasmid prior to its transfer to a recipient cell^6^. Two putative hexameric ATPases, TcpA and TcpF, are suggested to drive DNA coupling and pumping of a DNA-relaxase complex into the recipient cell^7–9^. Two other essential components of the transfer apparatus are the membrane spanning proteins TcpD and TcpE, both of which remain to be functionally characterized^10^. Finally, TcpG is a peptidoglycan hydrolase that is postulated to function by enabling a channel to be formed from the donor to the recipient cell^4^. The remaining three proteins, TcpB, TcpI and TcpJ, do not appear to be required for transfer.

We recently identified the pCW3 origin of DNA transfer site (*oriT*) as a 150 bp region that is located upstream of the *tcpM* gene and is nicked by the relaxase TcpM to form the relaxosome^6^. However, in vitro the nicking activity of TcpM does not appear to be sequence specific. Given this finding, we hypothesized that conserved genes located upstream or downstream of the *oriT* site may encode accessory proteins that provide the specificity for relaxosome formation. Here we demonstrate that one of these genes, *tcpK*, which is located immediately upstream of the *oriT* site (Fig. 1a) and encodes a conserved protein of unknown structure and function, is important for DNA transfer and represents a new component of the conjugative machinery.

## Results

### TcpK is required for efficient transfer of pCW3

We identified several conserved genes upstream of the *oriT* site of pCW3, one of which, *tcpK* (previously designated as *pcw328*^7^), encodes a putative 102 amino acid protein of unknown function. To determine whether TcpK was involved in conjugative transfer, the *tcpK* gene on pCW3 was inactivated using TargeTron (TT) technology. A *tcpK::TT* insertion mutant was selected using erythromycin resistance and confirmed by PCR and Southern hybridization analysis (Supplementary Figure 2). The mutant, JIR13063, was tested in mixed plate matings and showed a significant (4 log) reduction in transfer frequency compared to wild-type pCW3 (Fig. 1b). Introduction of the wild-type *tcpK* gene in *trans* restored transfer in the *tcpK* mutant back to wild-type levels. These results provide evidence that *tcpK* represents a novel and hitherto uncharacterized component of the conjugative machinery.

### TcpK is a winged helix-turn-helix transcription factor

TcpK has no sequence similarity to any protein of known structure or function. Accordingly, we set out to characterize the role of this molecule by determining its X-ray crystal structure. The 2.5 Å structure of TcpK (Fig. 1c) was determined by single anomalous diffraction (SAD) using data collected on selenomethionine-labeled protein crystals (Protein Data Bank (PDB) code 5VFY, Supplementary Table 1).

Four identical TcpK molecules were present in the asymmetric unit, each comprising four α-helices and five β-strands. DALI searches^11^ revealed that TcpK has structural similarity with winged Helix-Turn-Helix (wHTH) transcriptional regulators (Supplementary Table 2, and shares closest similarity with the N-terminal domain of MukF (root mean square deviation (r.m.s.d.) 3.4 Å over 79 Cα Å; Fig. 1d), a component of the bacterial chromosome partitioning system^12^. Structural and sequence comparisons of TcpK with the MukF wHTH DNA binding domain reveal that TcpK is, however, considerably smaller (102 residues) than MukF (440 residues). TcpK is also a structural homolog of several MarR-like transcription factors. Most notably, TcpK contains a major deletion in the C-terminal region and lacks the domain responsible for the dimerization of MukF and other members of the MarR family.

### TcpK features an unusual dimer that is also formed in solution

Macromolecular interface analysis of the crystal structure using PISA^13^ suggests that despite lacking the canonical MukF-like or MarR-like dimerization domain, TcpK is still predicted to form a dimer. These calculations are based on non-crystallographic symmetry-related subunits. The proposed dimer interface involves 48 residues for a total surface area of 866 Å^2^. These interactions are predominantly mediated through helices H1, H3 and H4 (Fig. 1c). Interfacing residues in helix H1 consist of V1–N5, all of which contribute through an extensive network of hydrogen bonds and/or salt bridges. Residues A8 and K9 of helix H1 also contribute via non-bonded interactions. Surprisingly, the second helix of the dimer interface (helix H3), involving Y57, L58 and E61–W64, is predicted to be involved in DNA binding. Finally, helix H4 provides additional interfacing residues with K89, S91, S92, S94–S96, R98 and E102. The achieved Complex Formation Significance Score of 1.00 implies that the interface plays an essential role in dimer formation.

Given these data, we set out to determine the quaternary structure of TcpK in solution. We conducted sedimentation velocity experiments at protein concentrations of 12, 24 and 48 μM (Fig. 1e). The resulting continuous sedimentation coefficient (*c*(*s*)) distributions showed a single symmetrical peak with a modal sedimentation coefficient of approximately 2.3 S. This sedimentation coefficient corresponds to a molecular mass of 26.7 kDa, assuming a frictional ratio (*f*/*f*_0_) of 1.27 estimated from the fit to the data for 48 μM TcpK (Supplementary Figure 3a-c), and suggested a dimeric structure (dimer mass from sequence is 26.1 kDa). The theoretical sedimentation coefficients for the dimer observed in the crystal structure and the corresponding monomer of TcpK were 2.2 S and 1.4 S, providing further support for a dimeric structure in solution. The absence of concentration dependence on the sedimentation properties of TcpK indicates that the dimer is stable at these concentrations and that the dissociation constant is well below the concentration range tested.

We next constructed a series of substitution derivatives to investigate whether the dimer observed in solution corresponded to the dimer observed in the crystal structure (Supplementary Figure 4). Two derivatives were designed to disrupt the TcpK dimer interface. TcpK_Dimer_ encoded four alanine substitutions (D3A, N5A, E63A and K89A) that would be anticipated to interrupt hydrogen bonds and salt bridges between the TcpK monomers. A second protein, TcpK_Rep_, was engineered to produce repulsive charges at the dimer interaction sites and encoded the substitutions N5E and E63K. It was not possible to express soluble recombinant TcpK_Dimer_ in *Escherichia* *coli*. However, TcpK_Rep_ was successfully expressed and purified. Gel filtration studies suggested that this protein was primarily monomeric (Supplementary Figure 4d), a finding confirmed by sedimentation velocity experiments (Fig. 1f).

To examine the function of these derivatives in vivo, we performed conjugation experiments using the *tcpK* mutant JIR13063. The *tcpK*_Dime*r*_ and *tcpK*_Rep_ mutants were cloned into the complementation vector, pJIR3422, and introduced into the *tcpK* mutant for subsequent testing. Both *tcpK*_Dimer_ and *tcpK*_Rep_ restored the conjugation frequency to wild-type levels (Fig. 1b), which suggested that monomeric TcpK was functional in vivo when expressed under these in *trans* conditions.

### TcpK forms a complex with DNA from the pCW3 *oriT* site

Studies of other conjugation systems suggest that accessory proteins within relaxosome complexes can either bind the relaxase enzyme or bind directly to the plasmid in the vicinity of the *oriT* site. Our data revealed that TcpK was a distantly related member of the wHTH family of DNA binding proteins. However, structural comparisons between TcpK and its closest homologs revealed key differences that seemed inconsistent with a role in DNA binding. In particular, the dimerization mode was incompatible with the usual arrangement of wHTH proteins in the context of binding DNA. Most notably, the canonical helix (H3) responsible for interacting with DNA in other wHTH superfamily members was buried at the dimer interface involving helices H1, H3 and H4’, and was hence inaccessible. Given these findings and to see if TcpK may be an accessory DNA binding component of the pCW3 relaxosome, gel mobility shift assays were performed with the recombinant TcpK protein and the pCW3 relaxase enzyme TcpM, both of which were produced in *E*. *coli*.

**Fig. 2:**
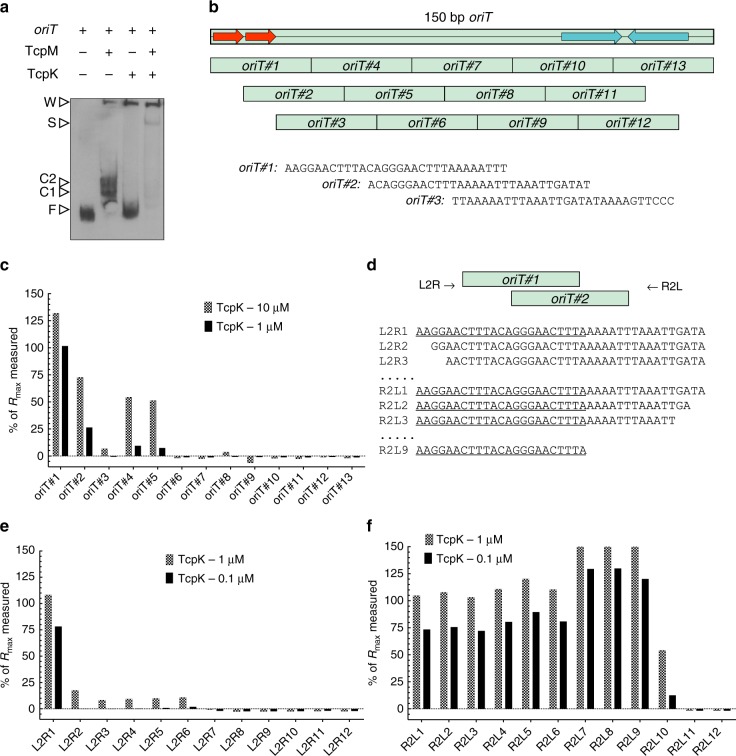
Identification of TcpK binding site. **a** Gel mobility shift assays with His_6_-TcpK. The DIG-labeled 150 bp *oriT* fragment was incubated alone, in the presence of 450 nM TcpM, in the presence of 750 nM TcpK or in the presence of 450 nM TcpM and TcpK (50 nM), as indicated. Reactions were separated on a 6% (w v^−1^) TBE acrylamide gel for 60 min at 200 V at 4 °C. Open arrows indicate free DNA (F), protein–DNA complexes (C), protein–DNA super-shifted complexes (S) and complexes in the wells (W). **b** SPR analysis of TcpK binding to *oriT*. The 150 bp *oriT* site of pCW3 was divided into overlapping fragments (*oriT#1–oriT#13*) each comprised of 30 bp with 20 bases overlap from one fragment to the next. The biotinylated single-stranded DNA (ssDNA) ReDCaT linker was immobilized on the surface of a Streptavidin (SA) chip to a surface density of 450 Resonance Unit (RU). **c** The binding of TcpK to the oligonucleotides was tested by SPR at protein concentrations of 10 μM (checked bars) and 1 μM (solid bars). The binding levels are expressed as a percentage of the theoretical maximum response (see Methods). **d** SPR-based mapping defines the TcpK binding site. A 39 bp fragment that encompassed *oriT#1 and oriT#2* was used as the initial test fragment (L2R1 and R2L1 for left to right and right to left, respectively). Subsequent oligonucleotides (L2R2 to L2R12 and R2L2 to R2L12) were incrementally shorter by 2 nt. TcpK binding to each oligonucleotide L2R (**e**) and R2L (**f**) was tested at protein concentrations of 1 μM (checked bars) and 0.1 μM (solid bars). The binding levels are expressed as a percentage of the theoretical maximum response

In the presence of TcpK alone, most of the 150 bp *oriT* fragment was observed in the unbound form, but a significant amount of DNA was detected in the wells, suggesting that TcpK can directly bind to *oriT* (Fig. 2a). The addition of TcpM, which we previously showed could bind to *oriT*^6^, resulted in almost all of the DNA shifting to this large complex, and virtually no free *oriT* DNA was observed. In addition, we observed a super-shifted band of increased size compared to TcpM binding to *oriT* alone.

These data suggest that TcpK alone could form a high molecular weight complex with the *oriT* DNA and supports the hypothesis that TcpK binds *oriT* in the vicinity of TcpM. However, we were not able to detect any interaction between the TcpK and TcpM proteins by chemical crosslinking.

### Identification of the DNA sequence bound by TcpK within* oriT*

Surface plasmon resonance (SPR) was used to identify the precise binding site of TcpK within the *oriT* site of pCW3. The 150 bp *oriT* region was divided into 13 overlapping 30 bp fragments (Fig. 2c) that were tested for their ability to bind to purified TcpK. The results (Fig. 2c) indicated that TcpK bound most effectively to the *oriT#1* fragment, with a binding level close to the maximum theoretical value (*R*_max_) for the lowest protein concentration. The level of TcpK binding to the *oriT#2–#13* fragments was considerably lower, in particular for *oriT#3* and *oriT#6–#13* where the values were close to the baseline, indicating that no significant binding was detected. To further compare the binding levels, steady-state affinity measurements were performed on *oriT#1–#3* (Supplementary Figure 5) and *K*_D_ values of 521 nM and 2.58 μM were obtained for *oriT#1* and *oriT#2*, respectively. No *K*_D_ value could be determined for *oriT#3* under similar experimental conditions. Accordingly, we concluded that the binding site for TcpK was restricted to *oriT#1* and *oriT#2*, which encompasses the first 40 nucleotides of the minimum *oriT* site required for conjugative transfer.

To identify the precise boundaries of the binding site, a mapping experiment was performed using the same SPR methodology^14^ starting with an oligonucleotide that encompassed the first 39 bp of the *oriT* site. Successive fragments for this experiment were each 2 bp shorter relative to one another (Fig. 2d). A total of 24 fragments were synthesized (12 for each boundary) and the binding of TcpK to each of them was monitored. Deletion of the first two bases at the 5’ end results in a total loss of binding (Fig. 2e), indicating that these bases are important for interaction with TcpK. At the 3’ end, however (Fig. 2f), TcpK displayed a consistently high level of binding (close to 100% *R*_max_) up to the 10th fragment, where the binding level dropped to 55% *R*_max_. In summary, this cut-down approach allowed us to identify the minimal sequence required for TcpK binding *oriT*_min23_ 5’-AAGGAACTTTACAGGGAACTTTA. This sequence contains a tandem repeat of GGAACTTTA (Fig. 2d) that will now be referred to as a TcpK box.

### TcpK exhibits a novel DNA binding mode for a wHTH domain

**Fig. 3:**
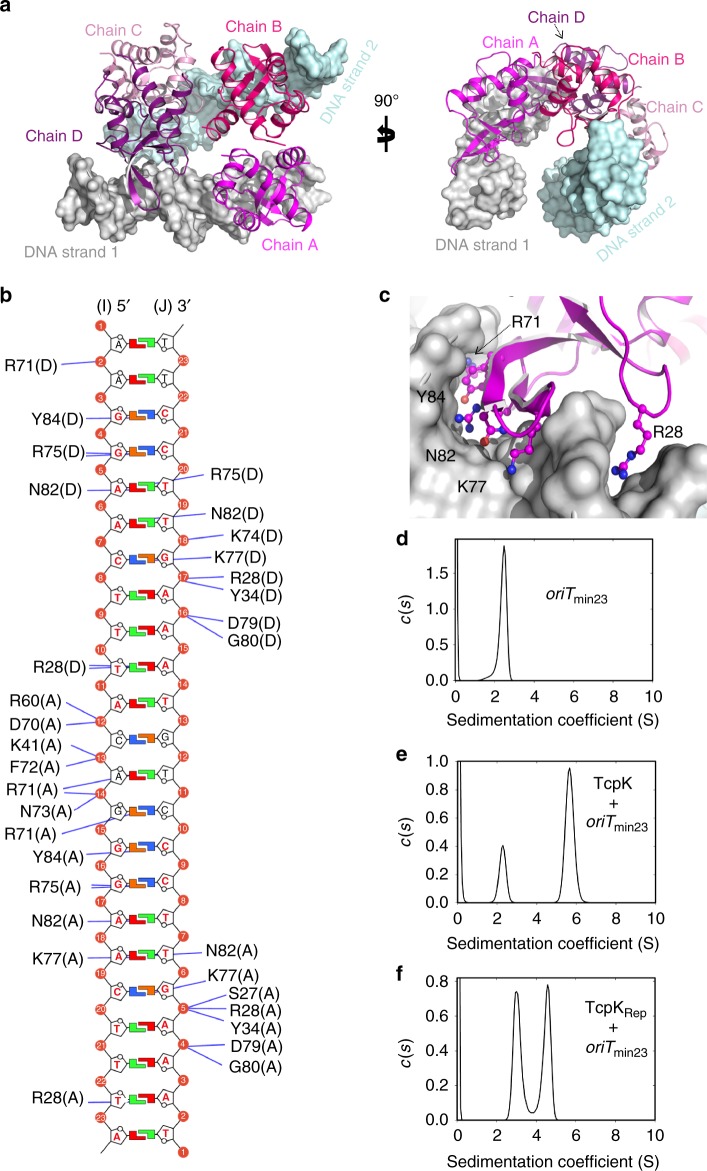
The crystal structure of TcpK in complex with *oriT*_min23_. **a** Ribbon representation of the overall structure of the complex. For clarity, only one half of the asymmetric unit is represented. Two orthogonal views along the vertical axis are presented. Each TcpK monomeric subunit is colored differently and DNA molecules are represented as surface structures. **b** Schematic representation of the contacts between TcpK and the DNA molecule. The respective chain IDs are presented in parentheses. For clarity, only hydrogen bond interactions are depicted and colored in blue, letters corresponding to the TcpK box nucleotides are colored in red. **c** Closer view of the DNA binding region. Selected residues of TcpK making hydrogen bond interactions with the DNA are represented as sticks. **d** Continuous sedimentation coefficient (*c*(*s*)) distribution for 2 μM *oriT*_min23_ and **e** for 24 μM TcpK in the presence of 12 μM *oriT*_min23_ calculated from the best fit to sedimentation data shown in Supplementary Figure 3d, e. **f** Continuous sedimentation coefficient (*c*(*s*)) distribution for 24 μM TcpK_Rep_ in the presence of 12 μM *oriT*_min23_ calculated from the best fit to the sedimentation data shown in Supplementary Figure 3g

To further understand the structural basis for TcpK binding to its nucleotide target, we determined the 2.8 Å crystal structure of the protein in complex with the tandem repeat double-stranded DNA (dsDNA) sequence identified by SPR (Supplementary Movie 1; PDB code 5VFX Supplementary Table 1 for data collection and refinement statistics). The asymmetric unit of the crystal contained four TcpK dimers (termed A:B, C:D, E:F, G:H) bound to four 23 bp dsDNA molecules. The structure was refined without non-crystallographic symmetry (NCS) restrains to final *R*_work_ and *R*_free_ values of 19.8 and 23.3%. Pairwise superposition of the eight protein chains (chain A–chain H) of the asymmetric unit indicate that these subunits are virtually identical (Global r.m.s.d. of 0.22–0.55 Å). Thus, initial description of the structure here is limited to one of the two TcpK/DNA complexes (i.e., A:B/C:D with two 23 bp molecules of DNA; Fig. 3a, b).

Superposition of TcpK to its DNA-bound form does not reveal any significant structural changes to the protein (Global r.m.s.d. of 0.40–0.9 Å). In addition, the dimerization interfaces are maintained and the expected DNA recognition helix is involved in dimer stability. However, the DNA molecules have undergone a subtle, but noticeable, twisting induced by TcpK binding (Supplementary Figure 6). A deeper analysis of the DNA structures with CURVES+^15^ and w3DNA^16^ show that the average helical twist is 35.22° ± 4.65 and the average rise per base pair is 3.17 Å ± 0.37. These values are close to that of an ideal 23 bp free DNA (36.00° ± 1.01 and 3.38 Å ± 0.01 respectively). However, the minor and major widths deviate significantly from ideal values in order to accommodate the protein (Supplementary Table 3). The minor groove is narrower in the first half of the molecule, while both minor and major grooves are on average more than 2 Å wider for the second TcpK box. This finding highlights a dissymmetry in the binding sites that may explain the slight bend of the DNA molecule, which is also reflected by the large deviation from standard values of the inclination angles at the center of the molecules (Supplementary Table 3).

Unexpectedly, each TcpK dimer bridges across two strands of DNA. This result is in contrast to any other known wHTH protein, where the dimer arrangement permits interaction through the canonical helix with palindromic sequences on the same DNA strand. In this context, analysis of the TcpK structure reveals that there is virtually no interface between TcpK monomers located on the same dsDNA molecule (i.e., chains A:D and B:C of Fig. 3a; PISA analysis suggests a complex significance score of 0).

Other researchers have reported that there are three distinct binding modes used by wHTH proteins to interact with DNA^17^. The canonical binding mode, widely used by MarR-like proteins, involves the recognition helix inserting into the major groove of the DNA (Supplementary Figure 7a). In the second mode, used by RFX1, the recognition helix interacts with the minor groove and the wing inserts into the major groove (Supplementary Figure 7b). The last mode of interaction, which is used by PCG2-DBD, involves the wing inserting into the minor groove and the helix interacting with the major groove (Supplementary Figure 7c).

Comparison of the TcpK/DNA complex with these wHTH/DNA assemblies reveals that TcpK interacts with DNA through a novel binding mode. Here the β-wing (β3–β4) sits within the major groove (Fig. 3c and Supplementary Figure 7d) while the canonical helix (H3) only makes a single DNA contact through R60 to the phosphate backbone. Additional residues making contact with the DNA are all located outside the canonical helix. These interactions are through the loops N22-K31 and E69-N73, and, in particular, the sidechain of R28, which stretches into the minor groove. Additionally, the protein makes hydrogen bond interactions with the inter TcpK box bases (-CAG-). For example, R71 makes contacts via Nε, NH1 and NH2 with the adenosine (A13/N7) and the guanosine (G14/N7 and G14/OP2) respectively (Fig. 3b). In addition, K41, D70, F72 and N73 interact with the phosphate backbone.

### Interacting TcpK boxes are spaced three nucleotides apart

**Fig. 4:**
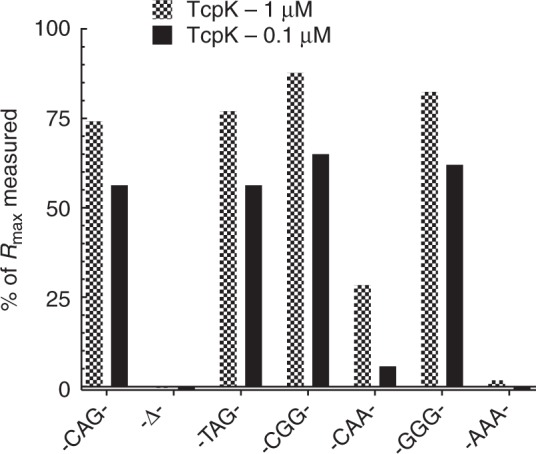
Binding analysis of TcpK binding to inter TcpK box linker sequence nucleotide derivatives. The 23 bp DNA sequence identified from the SPR mapping experiment has -CAG- as TcpK box linker sequence. Nucleotide substitutions at the 3-nt linker region are indicated on the graph and -Δ- is a deletion of all three bases. TcpK binding to each fragment was tested with protein concentrations of 1 μM (checked bars) and 0.1 μM (solid bars). The responses are expressed as a percentage of theoretical *R*_max_

The asymmetric unit of the TcpK–TcpK box co-crystal contains four TcpK dimers bound to four 23 bp dsDNA molecules (Movie 1). The crystal structure of the TcpK–TcpK box complex not only provided insights into the binding mode and the stoichiometry, but it also suggested that the three bases (-CAG-) located between the TcpK boxes may play a key role (Fig. 3b). The significance of these nucleotides in the context of protein/DNA interactions was therefore investigated using SPR. The 23 bp DNA fragment was used as the starting oligonucleotide for these experiments and substitutions made at the three nucleotides in the linker region. The removal of all three bases results in a total loss of binding (Fig. 4). Substitution of -CAG- with a -GGG- linker had no effect on the magnitude of the binding of TcpK. In contrast, substitution of -CAG- with -CAA- induced a significant reduction in the binding of TcpK (~25% *R*_max_) and no binding was observed for an -AAA- mutant. Consistent with these findings, the crystal structure of the complex reveals that TcpK interacts with the last base of the triad through hydrogen bonding interactions with R71.

### The quaternary structure of TcpK/DNA complex in solution

Subsequently, we investigated whether the interactions observed in the crystal structure corresponded to the TcpK–TcpK box complex made in solution. Sedimentation velocity analysis of *oriT*_min23_ (Supplementary Figure 3d) provided a *c*(*s*) distribution comprising one peak with a modal sedimentation coefficient of 2.5 S (Fig. 3d), consistent with a 23 bp dsDNA fragment. To confirm the stoichiometry of the TcpK–*oriT*_min23_ complex, sedimentation velocity experiments were conducted at a TcpK:*oriT*_min23_ molar ratio of 2:1 (Supplementary Figure 3e). The resulting *c*(*s*) distribution (Fig. 3e) comprised two peaks with modal sedimentation coefficients of 2.3 S, likely corresponding to excess free TcpK dimers, and 5.7 S, indicating formation of a complex. The baseline separation of the two peaks indicates that these species were sedimenting independently and that the positions of the peaks accurately reflected the sedimentation properties of each component. Modeling the sedimentation coefficient of the complex observed in the crystal structure (2 × TcpK dimers: 2 × *oriT*_min23_ duplexes, i.e., 2:2) provides a value of 5.9 S, in close agreement with the experimental sedimentation coefficient of the complex. In contrast, modeled sedimentation coefficients for stoichiometries of (2:1), (1:2) and (1:1) were 4.6, 4.7 and 3.5 S, respectively. Together, these data provide strong evidence that the quaternary structure of the TcpK–*oriT*_min23_ complex comprises two dimers of TcpK and two *oriT*_min23_ double-stranded molecules in solution.

### TcpK binding to DNA is required for conjugation in vivo

To validate the interactions made between TcpK and the DNA in the co-crystal structure, we generated two additional substitution derivatives. TcpK_DNA1_ was designed to disrupt the protein–DNA interaction observed from the co-crystal structure (R28A, R75A, N82A, Y84A). In the control derivative, TcpK_DNA2_ (D53A and Y57A), the substitutions were made to outwardly orientated residues in the helix that would normally interact with the major groove of the DNA in a canonical wHTH-like protein.

**Fig. 5:**
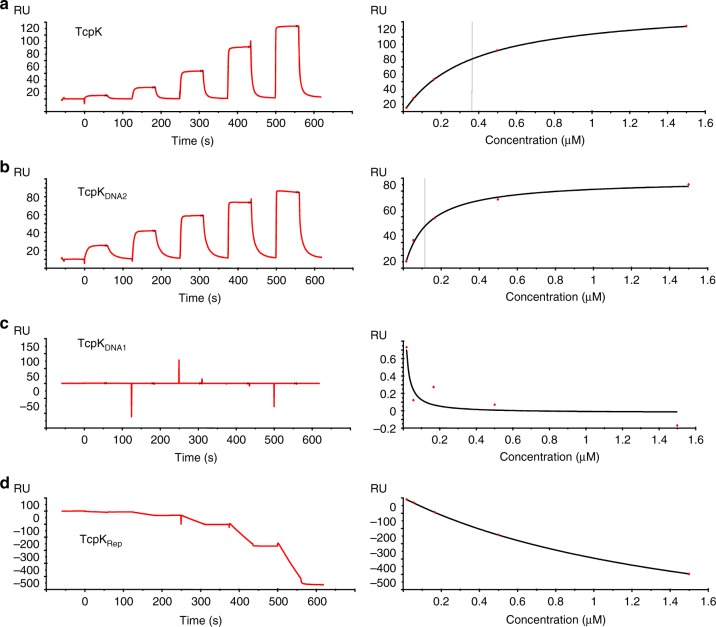
SPR binding analysis of TcpK binding to *oriT* fragments. Single-cycle steady-state affinity analysis of TcpK substitution derivatives binding to *oriT*_min23_. The left panels represent sensorgrams obtained for **a** TcpK, **b** TcpK_DNA2_, **c** TcpK_DNA1_ and **d** TcpK_Rep_ with protein concentrations of 18.5 nM, 55.6 nM, 166.7 nM, 500 nM and 1.5 μM. The right panels are the respective steady-state affinity fit graphs

Recombinant TcpK_DNA1_ and TcpK_DNA2_ were expressed in *E*. *coli*. Both proteins eluted by size exclusion at positions similar to that of the wild-type protein (Supplementary Figure 4b), suggesting that the dimer structure was maintained. SPR affinity measurements (Fig. 5c) revealed that TcpK_DNA1_ was no longer able to bind its DNA target, while TcpK_DNA2_ displayed an enhanced binding affinity to the 23 bp oligonucleotide (*K*_D_ of 120 nM, Fig. 5b) compared to the wild-type protein (*K*_D_ of 360 nM, Fig. 5a). In vitro DNA binding was further confirmed by fluorescence polarization experiments (*K*_D_ of 590 nM for TcpK and no binding was observed for TcpK_DNA1_, Supplementary Figure 8).

Next, the mutated genes were cloned into pJIR3422, introduced into the *tcpK* mutant, and tested in conjugation assays (Fig. 1b). The strain encoding the TcpK_DNA1_ protein did not restore conjugation and exhibited a conjugation frequency similar to that of the *tcpK* mutant. In contrast, the TcpK_DNA2_ derivative restored conjugation frequencies back to wild-type levels. Collectively, these data suggest that the TcpK residues identified in the crystal structure as being involved in DNA binding are important for TcpK function in vivo.

### Mutations abolishing TcpK dimerization still permit DNA binding

Our biophysical and structural data suggested that the dimeric nature of TcpK may play an important role in DNA recognition. Accordingly, we analyzed the interactions that the monomeric TcpK variant (TcpK_Rep_) makes with its DNA target. In SPR experiments, the sensorgram observed for TcpK_rep_ is consistent with a significant amount of protein binding to the reference flow cell (Fig. 5d); a finding that suggests a non-specific binding to either the dextran surface of the chip or the captured ReDCaT linker. To follow-up on this observation, sedimentation velocity experiments were conducted. A mixture of TcpK_Rep_:*oriT*_min23_ resulted in a *c*(*s*) distribution with two major peaks (3.0 and 4.6 S) (Fig. 3f). This distribution is consistent with the formation of multiple complex species and confirms that TcpK_Rep_ retains the ability to bind to DNA, albeit with an apparent loss of specificity. In regards to the latter point, we note that the canonical helix of the wHTH motif is exposed in the monomeric form of TcpK. In this context, we suggest that the absence of topological constraints originally provided by the dimer may contribute to the loss of DNA binding specificity.

## Discussion

In our previous work, we demonstrated that the unique TcpM protein functions as the pCW3 DNA relaxase^6^. However, these data also revealed that in contrast to other relaxases, TcpM possesses no detectable sequence specificity in vitro with respect to its ability to nick DNA. This finding led us to search for additional factors that may contribute such critical specificity to the pCW3 relaxosome complex.

In this study, we have identified TcpK, a protein encoded by a gene upstream of the *oriT* site and *tcpM*, as being a major factor required for the efficient conjugation of the pCW3 plasmid. TcpK represents the first protein encoded outside the current *tcp* locus to be shown to have a role in pCW3 transfer. We further demonstrated that TcpK is a bona fide DNA binding protein; it binds directly to the pCW3 *oriT* site and forms a super-shifted complex in the presence of the relaxase TcpM. These data support the idea that TcpK most likely functions as an accessory protein in the context of the pCW3 relaxosome.

TcpK has no significant sequence identity with any structurally or functionally characterized protein. Therefore, to understand its likely function we determined its X-ray crystal structure. Unexpectedly, we found that TcpK resembles a wHTH transcription factor. We showed that TcpK forms a dimer that is stable in solution. However, in contrast to conventional wHTH transcription factors, structural comparisons and bioinformatics analysis revealed that the arrangement of the two TcpK molecules in the dimer would not be predicted to be compatible with a canonical wHTH coordination of DNA molecules.

To complement these biophysical experiments, we determined the structure of TcpK in complex with the 23 bp region derived from *oriT*. These data revealed that TcpK binds DNA in a most unexpected fashion. In contrast to other wHTH proteins the β-wing of TcpK represents the primary point of contact with DNA and the canonical wHTH DNA binding helix is buried within the dimerization interface. In addition, and consistent with the results of our sedimentation velocity experiments, we found that each TcpK dimer bound to two TcpK boxes, but that these binding events take place in *trans* (i.e., to two different molecules of DNA).

To reconcile these in vitro data with the in vivo function of TcpK, we tested the ability of a range of different mutants to rescue conjugative transfer of a pCW3 *tcpK* mutant. The results showed that TcpK residues within the β-wing that are required for direct binding to the TcpK box are also required for efficient conjugative transfer, supporting the idea that TcpK is a bona fide DNA binding protein that likely functions in the context of the relaxosome. However, mutations shown to abolish TcpK dimerization in vitro had no detectable consequence on conjugation efficiency in vivo.

What are the implications of the integrated genetics and biophysical data for our understanding of the mechanism of transfer of members of the pCW3 family of conjugative antibiotic resistance and toxin plasmids? The *oriT* genetic data obtained previously^6^ and DNA binding data obtained here are in good agreement. We previously showed that the region containing the tandem repeat TcpK boxes in *oriT* are essential for efficient transfer. Here we demonstrate that TcpK is required for transfer and is capable of binding TcpK boxes.

Our in vitro data suggested that formation of the TcpK dimer was obligatory for specific DNA binding, and that this process involved the coordination of complexes that contain two DNA molecules. In contrast, the in vivo data revealed that monomeric forms of TcpK possess wild-type function in the context of conjugative transfer. The different nature of the DNA targets used in these studies may be the source of these apparently contradictory findings. In the bacterial cell the *oriT* target for TcpK binding is located on a large 47 kb supercoiled DNA molecule, whereas the in vitro studies were carried out using linear 23 bp fragments. In addition, we cannot rule out the possibility that the TcpK dimers are functioning in another biological process that may or may not be related to conjugative transfer. Indeed, we note that the canonical helix that binds DNA in other wHTH proteins forms much of the dimerization interface in TcpK, and is therefore no longer able to interact with DNA. Such an interaction might therefore have served to help drive the evolution of this molecule as a specialized component tightly restricted so that it functions in the context of conjugative plasmid DNA.

In conclusion, we identify TcpK as a crucial new component of the pCW3 conjugative machinery. Our data suggest that its function is to decorate the origin of transfer of pCW3 and to interact specifically with sequences that are only present in this region of the plasmid. In doing so we suggest that TcpK most likely functions within the relaxosome and may contribute with other protein partners to achieve the proper recruitment of TcpM, a relaxase that lacks detectable sequence specificity in vitro.

## Methods

### Bacterial strains, genetics and molecular methods

Bacterial strains and plasmids are described in Supplementary Table 4. *C.* *perfringens* and *E*. *coli* strains were cultured as previously described^6^. Molecular and genetics methods, including gel shift experiments and conjugative matings, were as before^6^. The *tcpK* mutant was constructed by TargeTron mutagenesis. An intron insertion site between residues 153 and 154 in the antisense strand of the 309 bp *tcpK* gene was identified using the Intron Finder software^18^ and intron primers re-targeted to the *tcpK* gene by primer-mediated PCR mutagenesis, as previously described^19^. The 350 bp PCR product was cloned between the *Hin*dIII and *Bsr*GI sites in the clostridial TargeTron vector pJIR3562^19^ to generate pJIR4347, which confers thiamphenicol resistance. *C*. *perfringens* strain JIR4195 [JIR325(pCW3)]^20^ was transformed with pJIR4347 and thiamphenicol-resistant transconjugants plated onto medium containing erythromycin (5 μg mL^−1^) to select for integration of the group II intron. Potential mutants that were tetracycline and erythromycin resistant, but thiamphenicol sensitive, were analyzed by PCR and Southern hybridization to confirm insertion of the intron in the *tcpK* gene. The resultant *tcpK::*TT mutant was designated as JIR13063.

For complementation studies, the wild-type *tcpK* gene was amplified by PCR and then cloned into the *Bam*HI/*Asp*718 sites of the *E*. *coli–C*. *perfringens* shuttle vector pJIR3422^4,21^ to generate pJIR4349. The complementation plasmid was introduced into the *tcpK* mutant by electroporation. The presence of the complementation plasmid in the *C*. *perfringens* strain was confirmed by restriction endonuclease and sequence analysis after plasmid rescue in *E*. *coli*. Mutant derivatives of *tcpK* (Dimer, Repulse, DNA1 and DNA2) were synthesized with the required mutations (Genscript) and subcloned from pUC57 (Genscript) into pJIR3422 by digestion with *Bam*HI/*Asp*718, as for the wild-type gene. Each construct was introduced separately into the *tcpK* mutant via electroporation and conjugation experiments were conducted as before^22,23^. Complementation vectors were checked as for the wild-type gene to confirm the strain constructions.

### Expression and purification of recombinant proteins

The genes encoding *tcpK* and its variants were codon optimized (Genscript) for expression in *E coli*. They were cloned first into pUC57 and then subcloned into pGL12 between the *Eco*RI and *Bam*HI restriction sites^24^. This cloning resulted in the expression of proteins with an N-terminal hexa-histidine tag followed by a TEV (tobacco etch virus) protease cleavage site. C41(DE3)pLysS was used as the expression host. Cells were grown in 2YT at 37 °C until the OD_600_ reached 0.6. The cultures were transferred to 16 °C for 30 min, protein expression was induced by the addition of 1 mM isopropyl β-d-1-thiogalactopyranoside (IPTG) and growth was continued for 18 h at 16 °C. The culture was harvested by centrifugation at 5000 × *g*, the pellets were resuspended in buffer A (50 mM Tris HCl, 300 mM NaCl, 10 mM imidazole, 2 mM 2-mercaptoethanol, pH 7.4) and lysed by sonication for 6 × 30 s pulses at 10 mA with 30 s rest between the pulses. The lysate was loaded on to a 1 mL Hi-Trap TALON column (GE Healthcare) equilibrated with buffer A, then washed with buffer B (50 mM Tris HCl, 1 M NaCl, 20 mM imidazole, 2 mM 2-mercaptoethanol, 10% (w v^−1^) glycerol, pH 7.4) and the protein eluted in buffer C (50 mM Tris HCl, 500 mM NaCl, 250 mM imidazole, 2 mM 2-mercaptoethanol, 10% (w v^−1^) glycerol, pH 7.4). The fractions were diluted twofold in TEV Buffer (100 mM Tris HCl, 150 mM NaCl, 0.2 mM EDTA, pH 8.0) and then incubated overnight with the TEV protease at a 1:100 (w w^−1^) (TEV/protein) ratio. The cleaved product was then reverse purified on the 1 mL TALON column. The flow through was collected, concentrated and further purified by size exclusion chromatography on a Superdex 75 16/60 (GE Healthcare) equilibrated with buffer D (25 mM Hepes, 150 mM NaCl, pH 7.4). When protein was used for crystallization experiments, the peak fraction was buffer exchanged into buffer E (25 mM MES, 50 mM NaCl, pH 6.5) and further purified by cation exchange chromatography on a Hi-Trap S FF (GE Healthcare) column. The protein was eluted using a gradient obtained by mixing buffer E and buffer F (25 mM MES, 1 M NaCl, pH 6.5). Purified selenomethionine-labeled TcpK was obtained in a similar fashion, but the cells were grown in M9 media supplemented with 50 mg L^−1^ selenomethionine and the buffers were supplemented with 5 mM 2-mercaptoethanol. The production of TcpM and the gel mobility shift assays were performed as described previously^6^.

### Protein crystallization and X-ray data collection

All crystals were obtained by hanging drop vapor diffusion with a 500 μL reservoir volume. For TcpK alone, the drops consisted of 1.5 μL TcpK (10 mg mL^−1^), 0.2 μL phenol (0.1 M) and 1.3 μL of the reservoir (1 M Na citrate, 0.1 M Na cacodylate, pH 6.5). Selenomethionine-labeled TcpK was obtained by microseeding with the same reservoir conditions. The seed stock was obtained by crushing unlabeled TcpK crystals then diluting tenfold with the reservoir solution. The drop consisted of 1.5 μL labeled TcpK (10 mg mL^−1^), 0.5 μL seed stock, 0.2 μL phenol (0.1 M) and 1 μL of the reservoir. TcpK–*oriT*_min23_ crystals were grown with a reservoir containing 50% (v v^−1^) 2-methyl-2,4-pentanediol, 0.1 mM imidazole, pH 7.0. The drop consisted of 1.5 μL TcpK–*oriT*_min23_ and 1.5 μL of the reservoir. Oligonucleotides were purchased from Sigma and dissolved in water as a 4 mM stock solution. Equal volumes of complementary strands were mixed, heated to 95 °C for 5 min and cooled to room temperature. TcpK (0.6 mg mL^−1^) and the double-stranded *oriT*_min23_ (2 mM) were mixed at a protein/DNA molar ratio of 2:1. The complex was concentrated to 380 μM for crystallization. Crystals were harvested using a silicon loop (Mitegen), soaked in the reservoir solution supplemented with either 25% (w v^−1^) sucrose or 25% (w v^−1^) glucose, then flash cooled in liquid nitrogen. X-ray diffraction data were collected on the MX2 beamline at the Australian Synchrotron. The best TcpK native crystal diffracted to 2.5 Å and was prone to radiation damage. Intensities were recorded from two distinct areas of the same crystal (Supplementary Table 1) and used for the refinement. Selenomethionine TcpK protein crystals also were highly prone to X-ray radiation damage. Anomalous diffraction data were collected at the selenium edge from four different protein crystals to resolution between 3.0 and 3.2 Å on the MX2 beamline. From each crystal, a maximum of 90° of X-ray data were recorded. One of these crystals allowed collection of datasets at three different positions. Each set of X-ray data was processed using XDS^25^. The resulting intensity data from each dataset were analyzed using Rd plot^26^ generated from the software XDSSTAT for radiation damage analysis allowed selection of a number of frames for final data processing for each dataset. The seven datasets were scaled together using XSCALE^25^. The merging statistics of scaled data (Supplementary Table 1) indicated anomalous signal up to 4.5 Å resolution.

### Structure determination

The structure of TcpK was solved using the SAD phasing protocol of Auto-Rickshaw^27^ that included substructure determination using SHELXD^28^, heavy atom refinement using BP3^29^ density modification using PIRATE^30^ and initial model building using BUCCANEER^30^. Further phase improvement, model completion and partial model refinement were carried out using MRSAD protocol of Auto-Rickshaw^31^, which resulted in a model with an *R*_work_ of 40% and *R*_free_ of 45%. The model was used as a starting model for MR phasing in Auto-Rickshaw against the native dataset. Density modification, phase extension and fourfold NCS averaging were carried out using RESOLVE^32^ to 2.5 Å resolution and further model building was performed using BUCCANEER. This allowed 399 residues to be built in four fragments out of 408 residues in the asymmetric unit and 393 residues were sequenced automatically. Initial refinement of the model was carried out in the REFMAC5^30^ and resulted in 27.4% *R*_work_ and 32.7% *R*_free_. The structure was improved iteratively with manual model building in COOT^33^ and refinement in BUSTER-TNT^34^.

The structure of TcpK–*oriT*_min23_ was determined by Molecular Replacement in PHASER^35^ in two steps. The initial search was performed using the coordinates of a single chain of TcpK. Upon inspection of PHASER’s output and the crystal packing, the difference Fourier maps displayed positive density, especially in the vicinity of the β-loop. Since no additional TcpK molecules could be placed in the unit cell, the missing information was therefore attributed to the presence of DNA in the crystal. Following on from this partial solution, the coordinates of the protein were fixed and a new search performed with a 23 bp dsDNA fragment generated in Coot^33^. However, this procedure failed and the search was repeated using a shorter dsDNA fragment (10 bp), which yielded several partial solutions that subsequently failed PHASER packing tests. These imperfect solutions were carefully examined in Coot and PyMOL, and used to manually position the DNA molecules in the electron density map. After several cycles of rebuilding and refinements, the final model was deposited in the PDB with *R*_work_ and *R*_free_ 0.5values of 19.8 and 23.3% respectively. Final models were validated with Molprobity^36^ and deposited in the PDB under the accession numbers 5VFY and 5VFX.

### Surface plasmon resonance

SPR experiments were performed on a Biacore T200 (GE Healthcare) operated at 20 °C. All experiments were performed in duplicate and were based on the Re-usable DNA Capture Technology (ReDCaT)^14^. The 150 bp *oriT* region (24697–24846) of pCW3 was divided into 13 overlapping fragments (*oriT#1–oriT#13*; Fig. 2d). Each fragment comprised 30 bp with a consecutive overlap of 20 bases from one fragment to the next. These fragments were generated using the Perl Overlapping Oligo Producer (POOP) script^14^. The complementary ReDCaT sequence was positioned at the 3’ end of the reverse oligonucleotide. Oligonucleotides (Sigma) were annealed together by incubating at 95 °C for 10 min then cooling to 20 °C on the bench. The protein was prepared as described earlier and dialyzed against the running buffer, which consisted of 10 mM HEPES pH 7.4, 200 mM NaCl, 3 mM EDTA and 0.05% (w v^−1^) Tween 20. The biotinylated ReDCaT linker was immobilized on two flow cells (FC1 and FC2) of a Streptavidin (SA) chip (GE Healthcare) by pulse injections of a 100 nM solution at 5 μL min^−1^. The injections were stopped when the density on the surface reached approximately 450 Resonance Units (RU). The flow cells then were washed with three 60 s injections of the regeneration solution (1 M NaCl, 50 mM NaOH).

For the initial screening, test dsDNA at 500 nM was captured for 60 s at 10 μL min^−1^ on the active flow cell (FC2). The protein (10 μM or 1 μM) was flowed over the two surfaces at 30 μL min^−1^ with 60 s contact time and 300 s dissociation time. The chip then was regenerated by injection of the regeneration solution for 60 s. Stability values were recorded 10 s after the end of protein injection. The binding level of TcpK to each fragment was expressed as a percentage of the theoretical maximum response (Theoretical *R*_max_ = (molar mass TcpK/molar mass *oriT#n*) × RU ligand captured × stoichiometry × 0.78 and %*R*_max_ measured = (measured *R*/theoretical *R*_max_) × 100). As suggested before^14^ the absolute values are not important for the analysis, but instead it is the relative binding levels among the fragments that provide the most valuable information.

For the mapping experiments, the two hits (*oriT#1* and *oriT#2)* were combined to form a 39 bp oligonucleotide L2R1 (left to right, relative to the top strand). Subsequent oligonucleotides were then synthetized (L2R2–L2R12), each shorter by 2 nt relative to one another. To identify the second boundary, the same starting oligonucleotide was used. Hence, R2L1 (right to left) and L2R1 are equivalent. However, R2L1 is oriented in the opposite direction on the sensor chip. This procedure guaranteed that the protein was able to interact with the oligonucleotide regardless of its orientation on the sensor chip. It also confirmed that the ReDCaT linker did not compensate for the loss of nucleotides from the test fragment. Protein concentrations used for the mapping were 1 μM and 0.1 μM and the binding level was expressed in the same manner as for the initial screening experiment.

For the initial kinetic (multicycle kinetic) measurements, approximately 250 RU of DNA was captured and TcpK was flowed over both FC1 (RedCat linker) and FC2 (*oriT#1*, *oriT#2* or *oriT#3*) at protein concentrations of 125 nM, 250 nM, 500 nM, 1 μM, 2.5 μM, 5 μM and 10 μM with an another 1 μM sample re-injected at the end of the experiment as internal duplicate. Subsequent single-cycle kinetic experiments were performed with 50 RU of DNA captured on FC2 and protein concentrations 18.5 nM–1.5 μM TcpK, TcpK_DNA1_, TcpK_DNA2_ and TcpK_Rep_ (60 s contact time at 30 μL min^−1^).

### Analytical ultracentrifugation

Sedimentation velocity experiments were performed using an XL-I analytical ultracentrifuge (Beckman Coulter) equipped with ultraviolet–visible scanning optics. Buffer reference (400 μL; 20 mM HEPES, 150 mM NaCl, pH 7.4) and sample solutions (380 μL) were loaded into 12 mm double-sector cells with quartz windows, and centrifuged at 50,000 rpm (201,600 × *g*) and 20 °C using an An-60Ti rotor. Radial absorbance data were recorded at appropriate wavelengths in continuous mode. Sedimentation velocity data were fitted to a continuous sedimentation coefficient distribution (*c*(*s*)) model using SEDFIT^37^. The partial specific volume of TcpK (0.7436 mL g^−1^), buffer density (1.006 g mL^−1^), and buffer viscosity (1.031 cp) were calculated using SEDNTERP^38^. The partial specific volume^39^ used for *oriT*_min23_ was 0.54 mL g^−1^ and partial specific volumes for various TcpK/*oriT* complex stoichiometries were calculated from the weighted average of protein and DNA. The molecular weight of TcpK was calculated from sedimentation coefficients in SEDFIT using the frictional ratio obtained from the fit to the sedimentation velocity data for 48 μM TcpK. Theoretical sedimentation coefficients were calculated from the TcpK and TcpK/*oriT*_min23_ complex crystal structure coordinates by hydrodynamic modeling under experimental conditions described above using HYDROPRO^40^.

### Fluorescence polarization DNA binding

Fluorescence polarization DNA binding experiments were performed using a PHERAstar microplate reader (BMG Labtech). Alexa488 5’-labeled *oriT*_min23_ was purchased from Sigma and annealed with the complementary non-labeled strand. The experiment was performed at 20 °C in a final reaction buffer consisting of 10 mM HEPES pH 7.4, 150 mM NaCl, 3 mM EDTA, 0.05% (w v^−1^) Tween 20, 0.5% (w v^−1^) bovine serum albumin. The DNA was incubated at a final concentration of 5 nM with various protein concentrations into a final volume of 50 μL. Gain and focus adjustments were performed on a protein-free sample. Background subtracted mP with the fits are reported in Supplementary Figure 8.

## Electronic supplementary material

Supplementary Information

Description of Additional Supplementary Files

Supplementary Movie 1

## Data Availability

All relevant data supporting the findings of the study are available in this article and its Supplementary Information files. The coordinates are available in the Protein Data Bank under accession codes 5VFY and 5VFX. Additional data are available from the corresponding authors upon request.
