# Supplementary material for: Crystal structure of TcpK in complex with oriT DNA of the antibiotic resistance plasmid pCW3
Source: Nat Commun. 2018 Sep 13;9:3732. doi: 10.1038/s41467-018-06096-2 (PMC6137059; doi:10.1038/s41467-018-06096-2)
Supplement: Supplementary file 1 — Supplementary Information [file 41467_2018_6096_MOESM1_ESM.pdf]

Supplementary Information, Traore *et al.*

Crystal structure of TcpK in complex with *oriT* DNA of the antibiotic resistance plasmid pCW3

Supplementary Figure 1

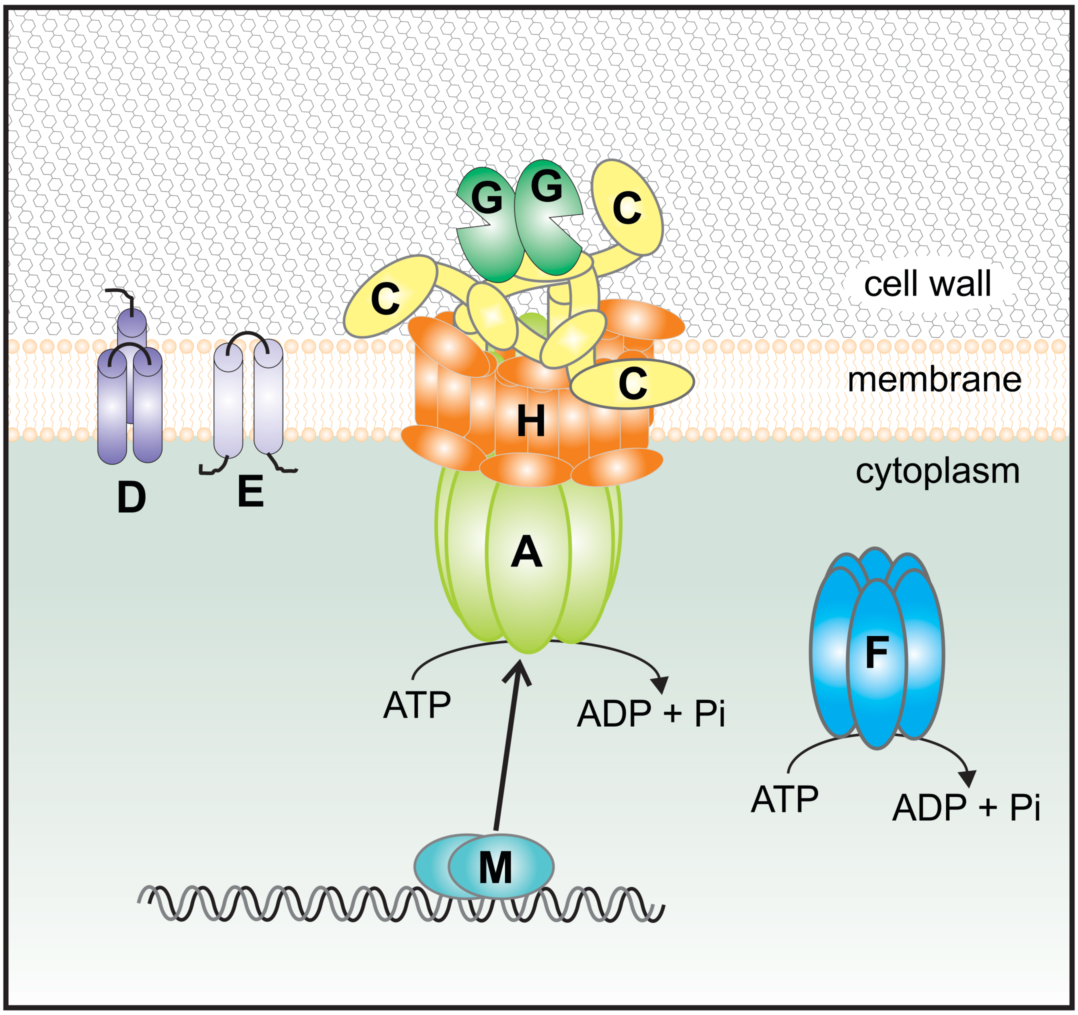

Model of the pCW3 conjugation apparatus. The core complex of the transfer apparatus is formed by the membrane proteins TcpH (orange shapes labelled with an "H") and TcpC (yellow shapes labelled with "C"). Other components include the novel relaxase TcpM (aqua ovals; "M"), two putative hexameric ATPases, TcpA (light green ovals; "A") and TcpF (Blue ovals; labelled "F"), two essential membrane spanning proteins TcpD (dark purple cylinders; "D") and TcpE (light purple cylinders; "E"). and the peptidoglycan hydrolase TcpG (dark green shape, labelled "G"). Protein components with a demonstrated biological interaction are indicated by overlap within the diagram, components with no detected protein-protein interactions are located separately.

Supplementary Figure 2

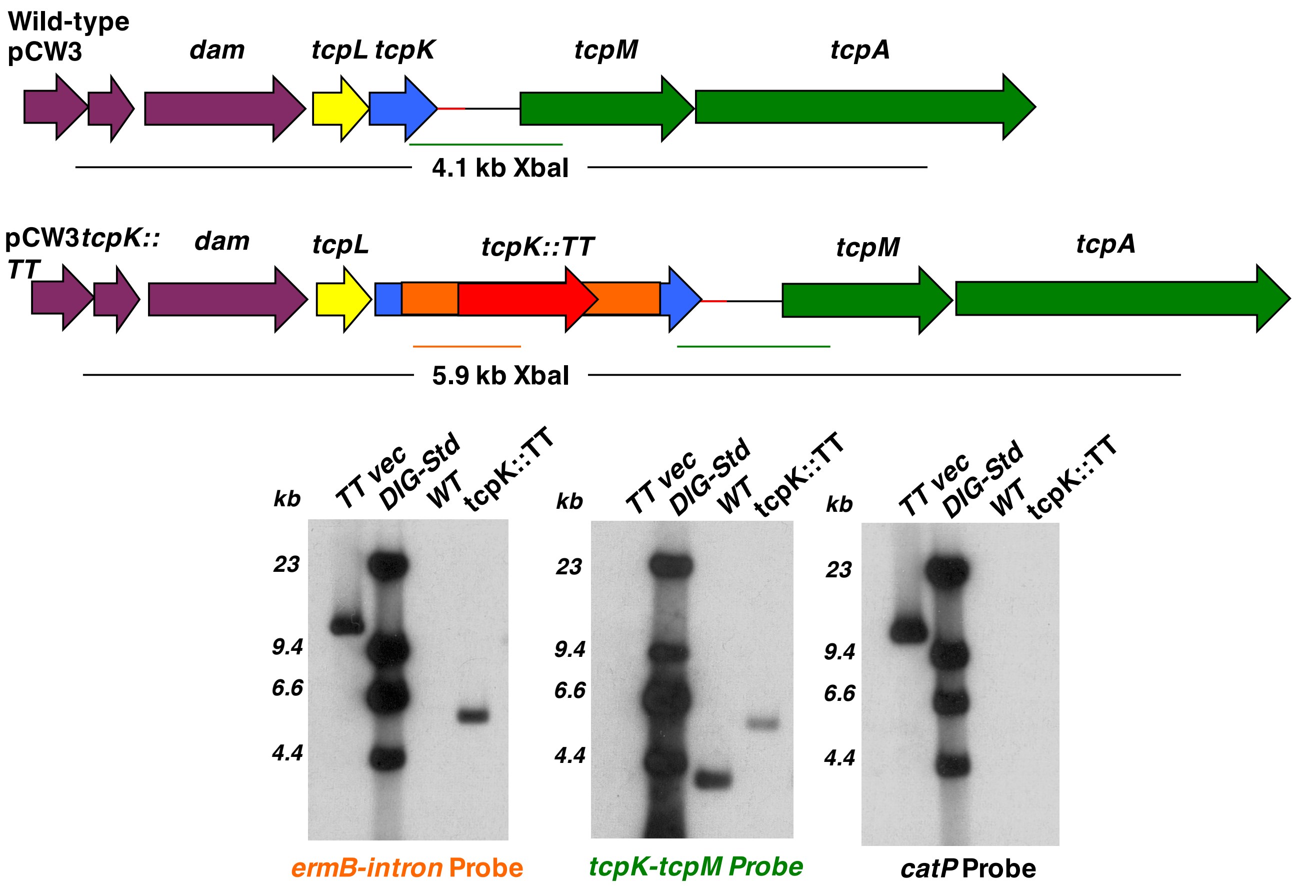

Confirmation of *tcpK* mutant. Southern hybridization analysis was carried out on *Xba*I-digested DNA from wild-type pCW3 and its isogenic pCW3*tcpK*::TT mutant. Probes were derived from the *ermB*-intron region (orange), the *tcpK-tcpM* region of pCW3 (green) and the *catP* gene from the targetron vector. Legend: TTvec (targetron vector, carries *ermB* and *catP* genes), DIG-Std (DIG-labeled λ*Hin*dIII DNA), WT (pCW3), *tcpK*::TT (pCW3*tcpK*::TT).

Supplementary Figure 3

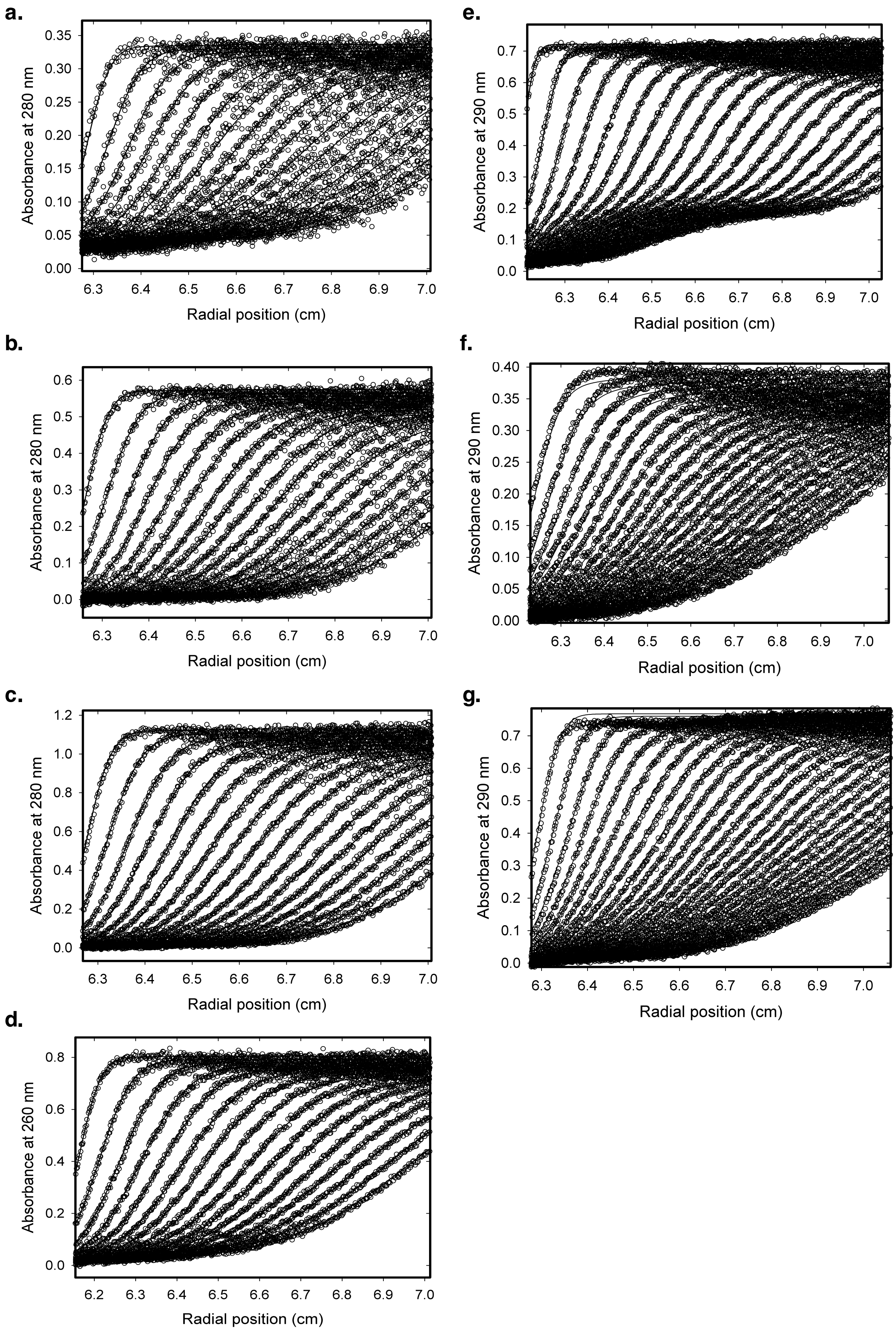

Sedimentation velocity data and fits for TcpK, *oriT*$_{min23}$, and TcpK / *oriT*$_{min23}$ complexes. Sedimentation velocity data and fits. Raw radial absorbance data (circles) are shown overlaid with the best fit to the continuous sedimentation coefficient [c(s)] distribution model (solid lines) for **(a)** 12 μM, **(b)** 24 μM, and **(c)** 48 μM TcpK. Radial absorbance scans collected at approximately 18 minute intervals during sedimentation are shown. **(d)** Raw radial absorbance data (circles) are shown overlaid with the best fit to the c(s) distribution model (solid lines) for 2 μM *oriT*$_{min23}$. Radial absorbance scans collected at 20 minute intervals during sedimentation are shown. **(e)** Raw radial absorbance data (circles) are shown overlaid with the best fit to the c(s) distribution model (solid lines) for 24 μM TcpK in the presence of 12 μM *oriT*$_{min23}$. Radial absorbance scans collected at 8 minute intervals during sedimentation are shown. Raw radial absorbance data (circles) are shown overlaid with the best fit to the continuous sedimentation coefficient [c(s)] distribution model (solid lines) for **(f)** 48 μM TcpK$_{Rep}$. Radial absorbance scans collected at 20 minute intervals during sedimentation are shown. **(g)** Raw radial absorbance data (circles) are shown overlaid with the best fit to the c(s) distribution model (solid lines) for 24 μM TcpK$_{Rep}$ in the presence of 12 μM *oriT*$_{min23}$. Radial absorbance scans collected at 8 minute intervals during sedimentation are shown.

Supplementary Figure 4

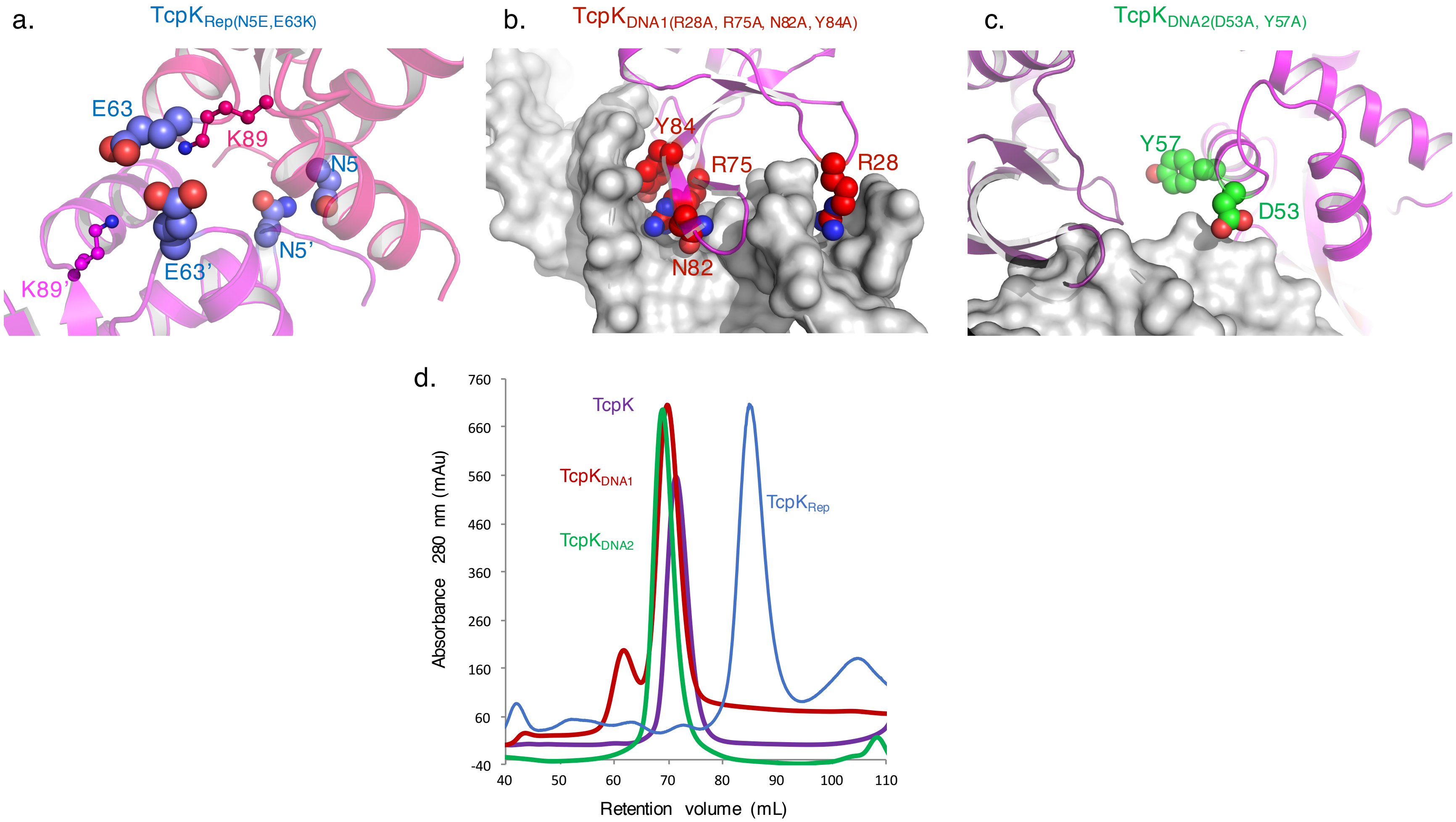

Design and purification of TcpK substitution derivatives. Substituted amino acids are depicted as sticks-balls on the crystal structures. **(a)** $TcpK_{Rep}$ (N5E, E63K) was designed to disrupt the dimer interface. The E63K substitution brings in additional positive charges at the interface around K89. **(b)** $TcpK_{DNA1}$ (R28A, R75A, N82A, Y84A) was designed to disrupt protein-DNA interactions and **(c)** $TcpK_{DNA2}$ (D53A, Y57A) is a control mutant containing substitutions in outwardly facing residues of the canonical DNA binding helix. **(d)** Purification of the recombinant proteins by size exclusion chromatography (S75 -16/60). The column was calibrated with molecules of known molecular sizes (GE Healthcare).

Supplementary Figure 5

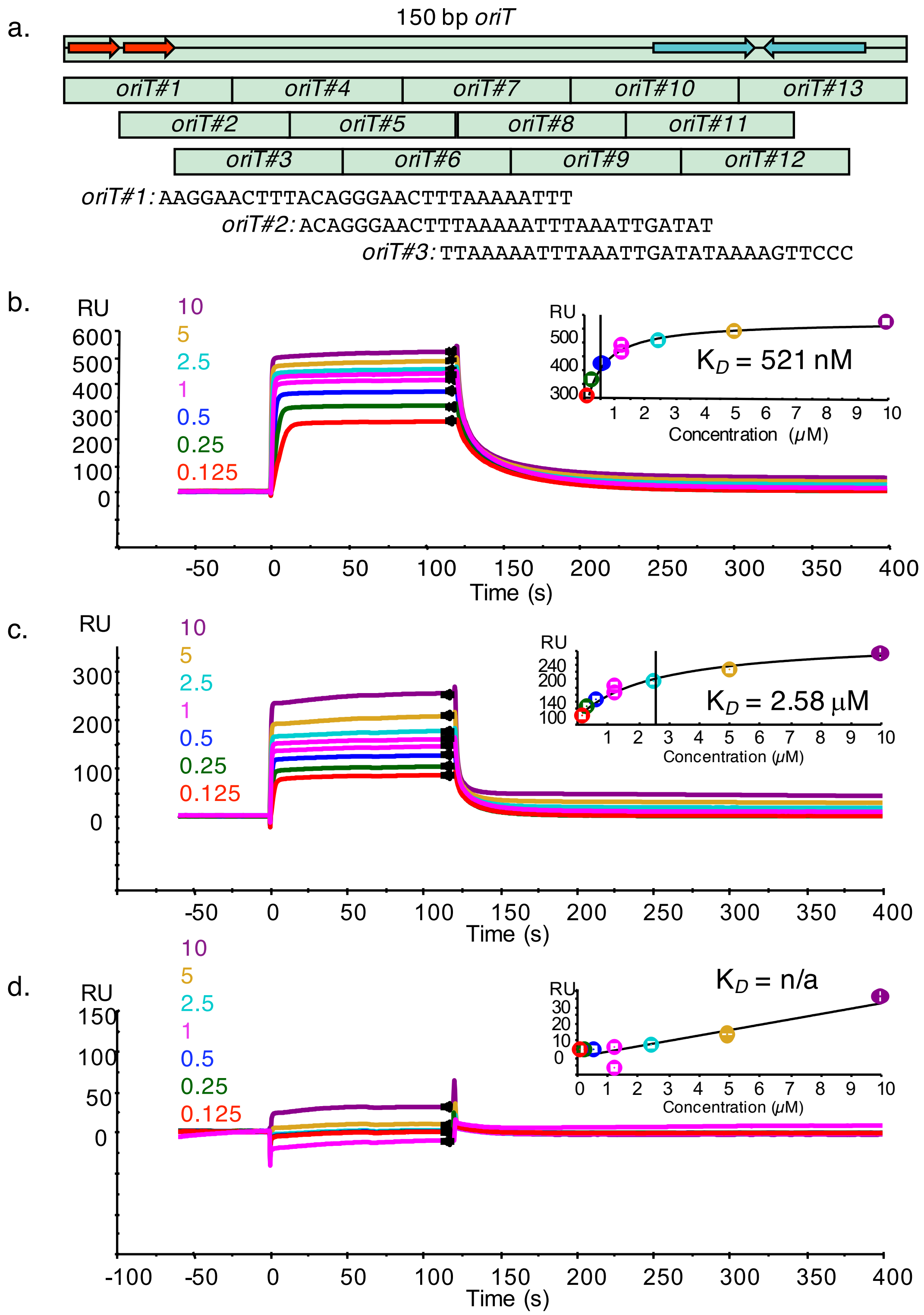

Steady state affinity binding analysis of TcpK binding to *oriT* fragments. (a) Sequences of the 30 bp fragments derived from the minimum pCW3 *oriT* region. Multicycle kinetic analysis of TcpK binding to *oriT#1* **(b)**, *oriT#2* **(c)** and *oriT#3* **(d)** are shown together with their respective steady state affinity fits. The protein concentrations used in this experiment ranged from 10 µM to 125 nM, as obtained by serial two-fold dilutions. TcpK concentration (µM) used with the corresponding sensorgrams concentration are coloured accordingly.

Supplementary Figure 6

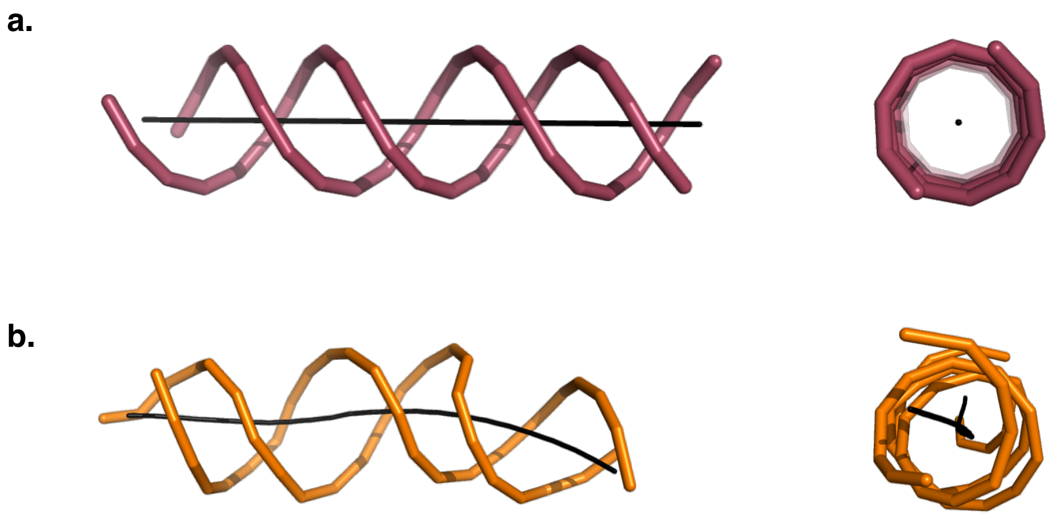

Protein induced bending and deformation of $oriT_{min23}$. Cartoon representation of $oriT_{min23}$ DNA without **(a)** and with TcpK bound **(b)** as seen in the crystal structure. Views along and through the axis of the helix are depicted. The axis of the helix is represented by the black line. Refer to Supplementary Table 3 for the groove and helical parameters of the molecules obtained from CURVES+[1] and w3DNA[2] .

Supplementary Figure 7

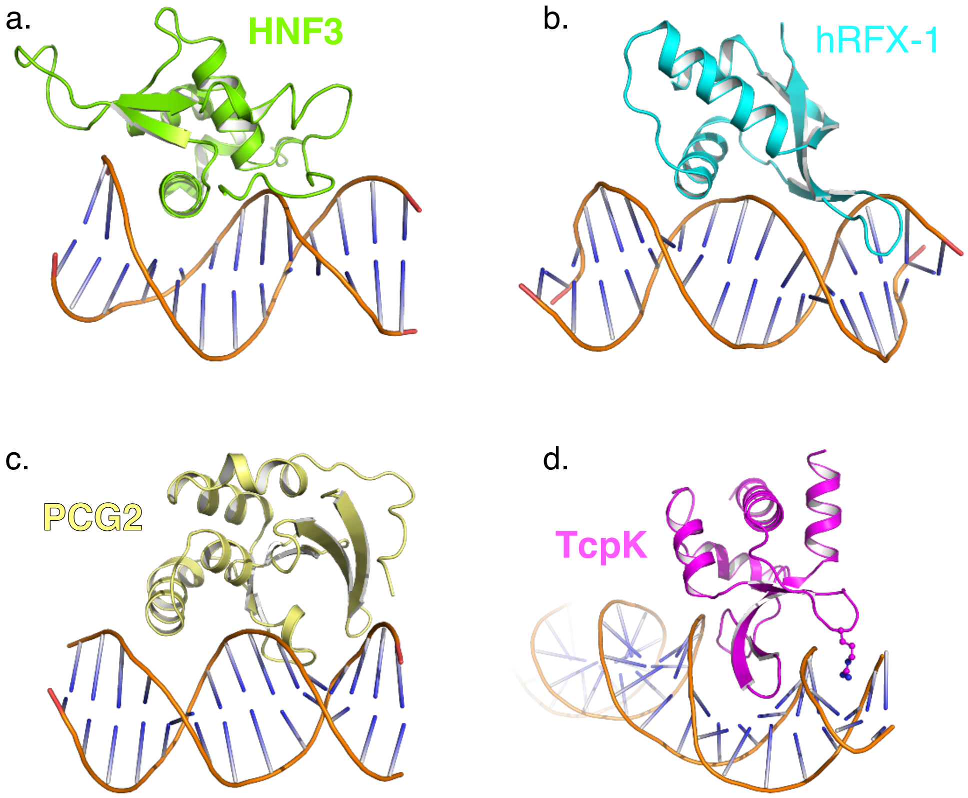

Description of binding mode used by wHTH proteins. (a) The canonical binding mode, widely used by HNF3 (PDB code 1VTB) and MarR-like proteins. The recognition helix inserts into the major groove of the DNA. **(b)** Second mode, used by hRFX1 (PDB code 1DP7), the recognition helix interacts with the minor groove and the wing inserts into the major groove. **(c)** The third mode of interaction described thus far and used by PCG2-DBD (PDB code 4UX5), involves the wing to insert in the minor groove and the helix to interact with the major groove. **(d)** The binding mode used by TcpK, the β-wing (β3-β4) sits in the major groove and the recognition helix is not required for DNA binding.

Supplementary Figure 8

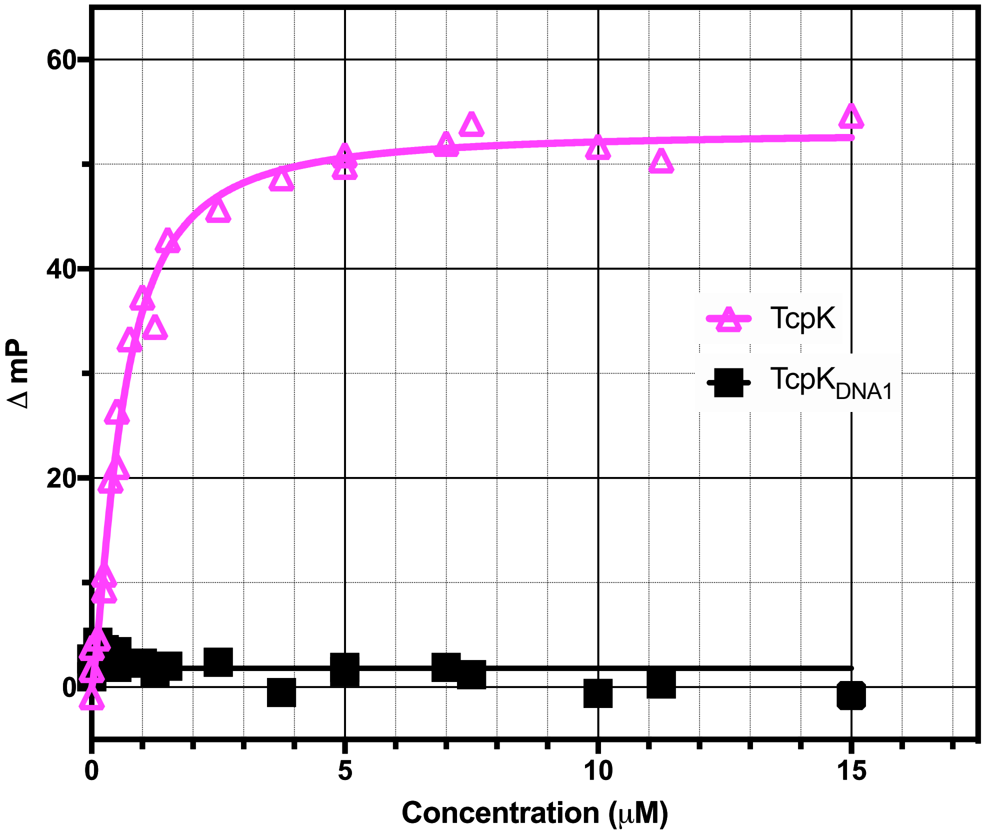

Fluorescence Polarisation (FP) binding isotherms of TcpK and TcpK$_{DNA1}$ to Alexa488 5' labelled *oriT$_{min23}$*. Raw FP data were background subtracted and plotted with the corresponding nonlinear regression fit. The binding isotherm for TcpK was fitted to specific binding model with the Prism 7 software. The fit for TcpK$_{DNA1}$ did not converge and plotted as a straight line for illustration. Experiments were performed in duplicate with internal replicates.

Supplementary Table 1: X-ray diffraction data collection and refinement statistics

	SeMet-TcpK	TcpK	TcpK-$oriT_{min23}$
**Data collection**			
Space group	$P\,2_1\,2_1\,2$	$P\,2_1\,2_1\,2$	$P\,2_1$
Cell dimensions			
a, b, c (Å)	82.41 119.08 51.60	80.56 120.59 52.89	69.50 109.89 122.78
α, β, γ (°)	90.0 90.0 90.0	90.0 90.0 90.0	90.0 93.02 90.0
Wavelength (Å)	0.9792	0.9537	0.9537
Resolution (Å)	48.26 - 3.22 (3.48 - 3.22)	48.44 - 2.49 (2.59 - 2.49)	41.48 - 2.81 (2.91 - 2.81)
R_{meas}	22.9 (72.6)	23.3 (95.1)	19.9 (245.8)
R_{pim}	5.3 (26.6)	8.8 (37.3)	16.5 (126.4)
$I / \sigma I$	11.8 (3.3)	10.8 (3.6)	6.5 (0.7)
CC (1/2)	0.990 (0.627)	0.984 (0.685)	0.992 (0.217)
Completeness (%)	80.5 (19.3)	98.9 (93.3)	98.4 (93.6)
Redundancy	16.7 (7.4)	6.3 (5.9)	3.7 (3.7)
Total reflections	117296 (2463)	118699 (11909)	166112 (15852)
Unique reflections	7009 (335)	18698 (2027)	44405 (4431)
**Refinement**			
No. reflections		18625	44345
R_{work} / R_{free}		18.1/23.3	19.8/23.3
No. atoms			
Protein		3410	6472
DNA		-	3752
Water		187	27
B-factors			
Protein		27.01	63.39
DNA		-	75.36
Water		34.57	41.98
R.m.s. deviations			
Bond lengths (Å)		0.010	0.010
Bond angles (°)		0.99	0.99

Supplementary Table 2: Top eight non redundant structural homologues identified by the DALI server

Protein	Function	Dali Z-score	Sequence Identity (%)	PDB	Residues used for SS matching	Reference
**MukF**	Chromosome partition protein	6.8	4	3EUH	24-115	3
**MepR***	Transcriptional Regulator	6.2	12	4LLN	28-106	4
**F93**	MarR-like viral protein	6.0	5	2CO5	5-96	5
**Topo-isomerase III**	Holiday Junction dissolution	6.0	8	4CHT	241-256 and 516-575	6
**MarR**	Transcriptional Regulator	6.0	12	1JGS	33-114	7
**SlyA***	Transcriptional Regulator	5.9	4	3Q5F	29-105	8
**CooA**	Transcriptional Regulator	5.9	9	2HKX	136-216	9
**RECQ1***	ATP dependent DNA helicase	5.9	7	4U7D	489-581	10

The function of the proteins, the Z-score and sequence identity with TcpK are reported. (*) denotes proteins crystallised with their respective DNA operator sequences.

Supplementary Table 3: Groove parameters and local helical parameters $oriT_{23min}$ DNA structures

	Step	Minor groove P-P (Å)		Major groove P-P (Å)		h-Rise (˚)		Inclination (˚)		Tip (˚)		h-Twist (˚)	
**1**	**AA/TT**	---	---	---	---	3.38	3.2	2.95	4.07	0	1.08	36	32.37
**2**	**AG/CT**	---	---	---	---	3.37	3.26	2.96	5.48	0.18	1.85	34.7	27.14
**3**	**GG/CC**	11.7	11.6	17.2	18.7	3.38	3.41	2.85	4	0.04	-1.35	36	33.52
**4**	**GA/TC**	11.7	10.3	17.2	18.8	3.39	3.25	2.84	-3.5	-0.12	1.11	37.32	40.77
**5**	**AA/TT**	11.7	9.7	17.2	18.9	3.38	3.14	2.95	-5.32	0	3.14	36	34.14
**6**	**AC/GT**	11.7	9.9	17.2	17.4	3.41	3.57	2.84	-5.79	0.12	3.44	37.66	34.33
**7**	**CT/AG**	11.7	9.5	17.2	17.4	3.37	3.49	2.96	5.84	-0.17	-0.37	34.69	39.2
**8**	**TT/AA**	11.7	9	17.2	17.3	3.38	3.28	2.95	-6.89	0	-3.76	36	40.84
**9**	**TT/AA**	11.7	11	17.2	17.6	3.38	3.1	2.95	-1.04	0	-2.99	36.01	35.69
**10**	**TA/TA**	11.7	14.5	17.2	16.8	3.37	3.09	2.95	4.62	0	7.1	35.65	36.91
**11**	**AC/GT**	11.7	15.4	17.2	15.9	3.41	3.21	2.84	-1.95	0.13	2.72	37.66	32.6
**12**	**CA/TG**	11.7	14.9	17.2	19.9	3.35	2.88	2.96	20.3	-0.18	-8.13	34.35	36.63
**13**	**AG/CT**	11.7	14.5	17.2	22.1	3.37	2.54	2.96	18.71	0.17	-3.16	34.68	31.23
**14**	**GG/CC**	11.7	14.7	17.2	20	3.38	1.93	2.85	32.87	0.04	-3.89	36.01	25.88
**15**	**GG/CC**	11.7	14.8	17.2	18.8	3.38	3.18	2.85	1.18	0.04	8.33	36.01	35
**16**	**GA/TC**	11.7	13.9	17.2	17.4	3.39	3.09	2.84	-2.69	-0.13	1.13	37.31	34.51
**17**	**AA/TT**	11.7	12.1	17.2	19	3.38	3.07	2.95	-1.31	0	-0.7	36	33.12
**18**	**AC/GT**	11.7	10.3	17.2	18.4	3.41	3.65	2.84	-7.25	0.13	0.75	37.67	36.19
**19**	**CT/AG**	11.7	9.5	17.2	17.6	3.37	3.63	2.96	6.25	-0.18	-2.96	34.69	34.61
**20**	**TT/AA**	11.7	9.5	17.2	17.2	3.38	3.25	2.95	-5.54	0	-0.82	36	40.28
**21**	**TT/AA**	---	---	---	---	3.38	3.18	2.95	-4.22	0	-3.89	36	32.66
**22**	**TA/TA**	---	---	---	---	3.37	3.39	2.95	3.88	0	-7.31	35.65	47.19
	**ave.**	11.70	11.95	17.20	18.29	3.38	3.17	2.91	2.8	0	-0.4	36	35.22
	**s.d.**	0.00	2.30	0.00	1.40	0.01	0.37	0.05	9.95	0.11	4.07	1.01	4.65

**Shaded values correspond to the TcpK bound DNA molecule.*

Supplementary Table 4: Plasmid and Strain table

Strain	Characteristics*	Reference
E. coli		
DH5α	General cloning strain. F$^-$ ϕ80 d*lacZ*ΔM15Δ(*lacZYA-argF*)U169 *endA1 recA1*	*Life Technologies*
C41(DE3)pLysS	BL21(DE3) with uncharacterised mutation(s). Protein expression studies	*11*
C. perfringens		
JIR4195	Strain 13 Rif^{R}Nal^R(pCW3)Tc^R	*12*
JIR4394	Strain 13 Str^{R}Chl^R	*13*
JIR13063	JIR4195(pCW3*tcpK*::TT), Tc^R Em^R	*This Study*
Plasmids		
pCW3	Conjugative, *C. perfringens* plasmid, Tc^R	*14*
pGL12	Modified pCOLD(IV) vector with His$_6$-TEV-maspin between NdeI/HindIII sites, Ap^R	*15*
pUC57	Cloning vector Ap^R	*Genscript*
pJIR3422	*E. coli-C. perfringens* shuttle vector, Cm^R	*16, 17*
pJIR3562	TargeTron mutatgenesis plasmid, P$_{gdh}$ group II intron, Cm^R	*18*
pJIR4347	pJIR3562 retargetted to interrupt pCW3 *tcpK*, Cm^R	*This Study*
pJIR4411	pJIR3422 *tcpK*$^+$ cloned Asp718/BamHI, Cm^R	*This Study*
pJIR4577	pJIR3422 *tcpK$_{dimer}$* cloned Asp718/BamHI, Cm^R	*This Study*
pJIR4579	pJIR3422 *tcpK$_{DNA2}$* cloned Asp718/BamHI, Cm^R	*This Study*
pJIR4580	pJIR3422 *tcpK$_{Rep}$* cloned Asp718/BamHI, Cm^R	*This Study*
pJIR4581	pJIR3422 *tcpK$_{DNA1}$* cloned Asp718/BamHI, Cm^R	*This Study*

*Antibiotic resistance (^{R}) abbreviations; Rif (rifampicin), Nal (naladixic acid), Tc (tetracycline), Str (streptomycin), Chl (potassium chlorate), Em (erythromycin), Ap (ampicillin), Cm (chloramphenicol).

Supplementary References

1. Lavery R, Moakher M, Maddocks JH, Petkeviciute D, Zakrzewska K. Conformational analysis of nucleic acids revisited: Curves+. *Nucleic acids research* **37**, 5917-5929 (2009).

2. Zheng G, Lu XJ, Olson WK. Web 3DNA--a web server for the analysis, reconstruction, and visualization of three-dimensional nucleic-acid structures. *Nucleic acids research* **37**, W240-246 (2009).

3. Woo JS*, et al.* Structural studies of a bacterial condensin complex reveal ATP-dependent disruption of intersubunit interactions. *Cell* **136**, 85-96 (2009).

4. Birukou I, Seo SM, Schindler BD, Kaatz GW, Brennan RG. Structural mechanism of transcription regulation of the Staphylococcus aureus multidrug efflux operon mepRA by the MarR family repressor MepR. *Nucleic acids research* **42**, 2774-2788 (2014).

5. Larson ET*, et al.* A winged-helix protein from Sulfolobus turreted icosahedral virus points toward stabilizing disulfide bonds in the intracellular proteins of a hyperthermophilic virus. *Virology* **368**, 249-261 (2007).

6. Bocquet N*, et al.* Structural and mechanistic insight into Holliday-junction dissolution by topoisomerase IIIalpha and RMI1. *Nat Struct Mol Biol* **21**, 261-268 (2014).

7. Alekshun MN, Levy SB, Mealy TR, Seaton BA, Head JF. The crystal structure of MarR, a regulator of multiple antibiotic resistance, at 2.3 A resolution. *Nat Struct Biol* **8**, 710-714 (2001).

8. Dolan KT, Duguid EM, He C. Crystal structures of SlyA protein, a master virulence regulator of Salmonella, in free and DNA-bound states. *J Biol Chem* **286**, 22178-22185 (2011).

9. Borjigin M, Li H, Lanz ND, Kerby RL, Roberts GP, Poulos TL. Structure-based hypothesis on the activation of the CO-sensing transcription factor CooA. *Acta crystallographica Section D, Biological crystallography* **63**, 282-287 (2007).

10. Pike AC*, et al.* Human RECQ1 helicase-driven DNA unwinding, annealing, and branch migration: insights from DNA complex structures. *Proc Natl Acad Sci U S A* **112**, 4286-4291 (2015).

11. Miroux B, Walker JE. Over-production of proteins in Escherichia coli: mutant hosts that allow synthesis of some membrane proteins and globular proteins at high levels. *Journal of molecular biology* **260**, 289-298 (1996).

12. Hughes ML*, et al.* Epsilon-toxin plasmids of Clostridium perfringens type D are conjugative. *J Bacteriol* **189**, 7531-7538 (2007).

13. Bannam TL, Teng WL, Bulach D, Lyras D, Rood JI. Functional identification of conjugation and replication regions of the tetracycline resistance plasmid pCW3 from Clostridium perfringens. *J Bacteriol* **188**, 4942-4951 (2006).

14. Rood JI, Scott VN, Duncan CL. Identification of a transferable tetracycline resistance plasmid (pCW3) from Clostridium perfringens. *Plasmid* **1**, 563-570 (1978).

15. Law RH*, et al.* The high resolution crystal structure of the human tumor suppressor maspin reveals a novel conformational switch in the G-helix. *J Biol Chem* **280**, 22356-22364 (2005).

16. Adams V*, et al.* Utility of the clostridial site-specific recombinase TnpX to clone toxic-product-encoding genes and selectively remove genomic DNA fragments. *Appl Environ Microbiol* **80**, 3597-3603 (2014).

17. Bantwal R*, et al.* The peptidoglycan hydrolase TcpG is required for efficient conjugative transfer of pCW3 in Clostridium perfringens. *Plasmid* **67**, 139-147 (2012).

18. Cheung JK*, et al.* The VirSR two-component signal transduction system regulates NetB toxin production in Clostridium perfringens. *Infect Immun* **78**, 3064-3072 (2010).
