# Supplementary material for: Crystal structure of TcpK in complex with oriT DNA of the antibiotic resistance plasmid pCW3
Source: Nat Commun. 2018 Sep 13;9:3732. doi: 10.1038/s41467-018-06096-2 (PMC6137059; doi:10.1038/s41467-018-06096-2)
Supplement: Supplementary file 2 — Description of Additional Supplementary Files [file 41467_2018_6096_MOESM2_ESM.pdf]

Description of Additional Supplementary Files

File Name: Supplementary Movie 1
Description: **The crystal structure of TcpK with tandem repeat TcpK boxes.** Cartoon representation of the asymmetric unit of the co-complex crystal. The movie presents a 360 º rotation around the vertical axis. The asymmetric unit contains four TcpK dimers coloured in purple, orange, red and green, and two 23 bp DNA molecules depicted in grey
